# Structural, Luminescent and Thermal Properties of Heteronuclear Pd^II^–Ln^III^–Pd^II^ Complexes of Hexadentate N_2_O_4_ Schiff Base Ligand

**DOI:** 10.3390/molecules23102423

**Published:** 2018-09-21

**Authors:** Barbara Miroslaw, Beata Cristóvão, Zbigniew Hnatejko

**Affiliations:** 1 Department of Crystallography, Faculty of Chemistry, Maria Curie-Sklodowska University, Maria Curie-Sklodowska sq. 3, 20-031 Lublin, Poland; 2 Department of General and Coordination Chemistry, Faculty of Chemistry, Maria Curie-Sklodowska University, Maria Curie-Sklodowska sq. 2, 20-031 Lublin, Poland; beata.cristovao@poczta.umcs.lublin.pl; 3 Department of Rare Earths, Faculty of Chemistry, Adam Mickiewicz University in Poznań, Umultowska 89b, 61-614 Poznań, Poland; zbychuh@amu.edu.pl

**Keywords:** 4d-4f complexes, palladium complexes, luminescence, Schiff base heteronuclear complexes

## Abstract

New Pd^II^–Ln^III^–Pd^II^ complexes of hexadentate N_2_O_4_ Schiff base ligand (H_4_L: *N*,*N*′-bis(2,3-dihydroxybenzylidene)-1,3-diamino-2,2-dimethylpropane) with Eu (**1**), Tb (**2**), Er (**3**) and Yb (**4**) ([Pd_2_Eu(H_2_L)_2_NO_3_](NO_3_)_2_∙2H_2_O∙2CH_3_OH **1**, [Pd_2_Ln(H_2_L)_2_H_2_O](NO_3_)_3_∙3H_2_O, where Ln = Tb **2**, Er **3**, [Pd_2_Yb(H_2_L)_2_H_2_O](NO_3_)_3_∙5.5H_2_O **4**) were synthesized and characterized structurally and physicochemically by thermogravimetry (TG), differential thermogravimetry (DTG), differential scanning calorimetry (DSC) and luminescence measurements. The compounds **1**–**4** are built of cationic heterometallic Pd^II^–Ln^III^–Pd^II^ trinuclear units. The palladium(II) centers adopt a planar square geometry occupying the smaller N_2_O_2_ cavity of the Schiff base ligand. The lanthanide(III) is surrounded by two Schiff base ligands (eight oxygen atoms) and its coordination sphere is supplemented by a chelating bidentate nitrate ion in **1** or by a water molecule in **2**–**4**. The complexes have a bent conformation along the Pd^II^–Ln^III^–Pd^II^ line with valence angles in the ranges of 162–171°. The decomposition process of the complexes results in mixtures of: PdO, Pd and respective lanthanide oxides Eu_2_O_3_, Tb_2_O_3_, Tb_4_O_7_, Er_2_O_3_, Yb_2_O_3_. The luminescent measurements show low efficiency intramolecular energy transfer only in the complex of terbium(III) (**2**).

## 1. Introduction

The studies on palladium complexes are mainly focused on their catalytic activity [1,2,3]. Pd is widely applied in fuel cells, electronics and the automobile industry, as well as in dental and jewelry alloys. The extensive usage of this metal contributes to waste production and environmental pollution emissions to water and soil [4]. Therefore, fluorescent sensors for the detection of palladium ions are necessary and luminescence of palladium complexes has become an important topic [5]. On the other hand, the coordination compounds containing Ln^III^ ions usually have quite long fluorescent life times, high quantum efficiency, sharp emission bands and large Stockes shifts. Therefore, the combination of Pd^II^–Ln^III^ may result in compounds with interesting properties.

In the Cambridge Structural Database (CSD) (version 5.39 with updates) [6], there are 74 unique crystal structures of heteronuclear Pd^II^–Ln^III^ coordination compounds. Most of them (29) are complexes of carboxylic or thiocarboxylic acids. Among the other ligands there are structures with arsenates (12), cyanides (11), pyridine and pyrimidine derivatives (10), phosphines (4) and others. The Schiff bases are known to easily form 3d-4f complexes with transition metal ions such as Ni^II^, Cu^II^ and Zn^II^ [7,8,9,10,11,12]. To conform the specific coordination stereochemical requirements of a palladium(II) ion, the structure of the organic ligand should provide a possibility to create a square planar coordination site. In the literature, there are nine homonuclear Pd^II^ crystal structures with azomethino ligands bearing a 1,3-propanediimino spacer, and among them, there is only one structure with a bi-compartmental Schiff base [13]. Equally important is the possibility to adapt the molecular conformation and the relative positon of two voluminous organic ligand molecules to additionally coordinate the lanthanide(III) ion. There is only one report in the CSD of a crystal structure of a heteronuclear Pd–Ln complex (Pd^II^–Sm^III^–Pd^II^). It is a complex of a bi-compartmental Schiff base ligand (2,2′-(cyclohexane-1,2-diylbis(iminomethylene))bis(6-ethoxyphenolate)), which was synthesized for the asymmetric Friedel–Crafts alkylation of pyrroles with nitroalkenes [14].

**Scheme 1:**
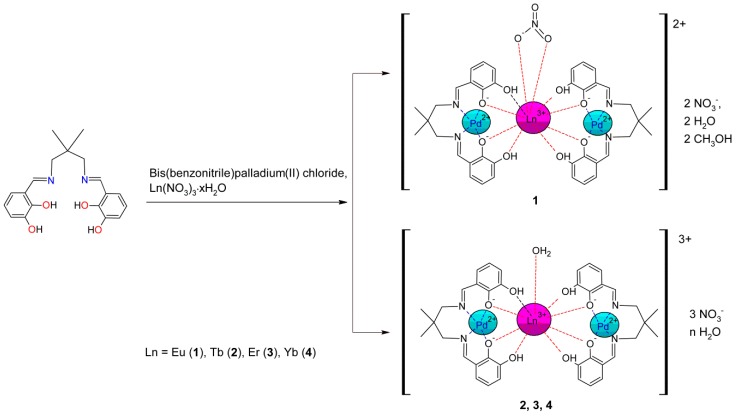
Synthesis of heterotrinuclear coordination compounds Pd^II^–Ln^III^–Pd^II^ (Ln = Eu (**1**), Tb (**2**), Er (**3**), Yb (**4**)) of *N*,*N*′-bis(2,3-dihydroxybenzylidene)-1,3-diamino-2,2-dimethylpropane (H_4_L).

To expand the current knowledge about heterometallic Ln(III) coordination compounds, we present a series of four structurally characterized Pd^II^–Ln^III^–Pd^II^ complexes with a hexadentate N_2_O_4_ ligand *N*,*N*′-bis(2,3-dihydroxybenzylidene)-1,3-diamino-2,2-dimethylpropane (H_4_L) (Scheme 1). Previously, we reported complexes of the H_4_L ligand with Cu^II^–Ln^III^–Cu^II^ metal ions [15]. The geometry of the coordination sphere around the pentacoordinated copper(II) showed a high degree of planarity of the N_2_O_2_ chelating cavity with low deviation of the Cu^II^ ion from the base plane. This prompted us to trial obtaining novel palladium(II) heteronuclear 4d-4f complexes. The crystal structures presented here enable us to better know the coordination preferences and stereochemistry of palladium(II) ions in combination with voluminous Schiff base ligands in heterometallic lanthanide(III) compounds. The thermal behaviour and photoluminescence properties of complexes **1**–**4** are also discussed.

## 2. Results

### 2.1. Crystal Structure

The crystal structure solution, refinement details and the selected molecular geometric data are given in Table 1 and Table S1 (in Supporting Materials).

The combination of a N_2_O_4_ hexadentate two-compartmental H_4_L ligand with a hard Lewis acid (Ln^3+^ ions) and a soft Lewis acid in form of a Pd^2+^ cation resulted in the formation of propeller-like heterotrinuclear coordination compounds.

**Figure 1:**
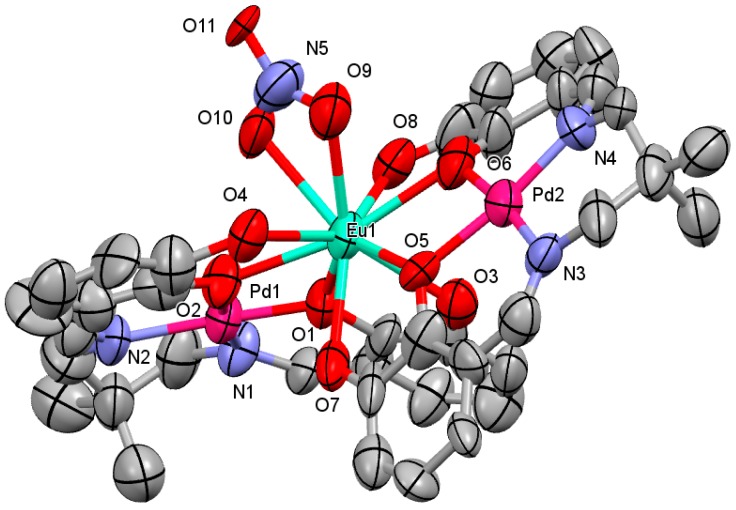
Molecular structure of the coordination unit in **1**.

Complex **1** (Pd^II^–Eu^III^–Pd^II^) crystallizes in the monoclinic space group *P*2_1_/*c*. The polyhedron around the ten-coordinated Ln(III) ion is formed by eight O atoms from two hexadentate ligand molecules and by two O atoms from the chelating bidentate nitrate anion (Figure 1). The coordination unit is cationic and two additional NO_3_^−^ anions are located in the outer coordination sphere. In the crystal structure, there are also two methanol and two water molecules.

**Figure 2:**
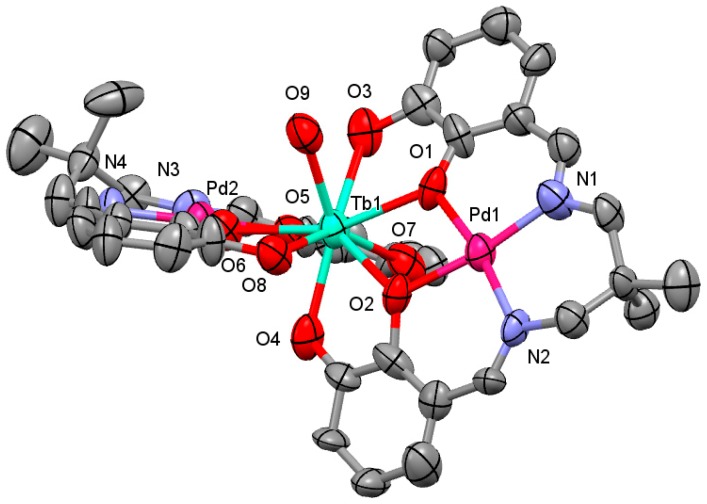
Molecular structure of the coordination unit in **2**.

Compounds **2** (Pd^II^–Tb^III^–Pd^II^) and **3** (Pd^II^–Er^III^–Pd^II^) are isostructural (space group *P*2_1_/*n*) (Figure 2). The lanthanide(III) cation has a coordination number of nine. Opposite to complex **1** in its coordination sphere, there is a water molecule instead of a bidentate nitrate ion. Three NO_3_^−^ ions are involved in intermolecular interactions with the coordination unit and with the three water molecules present in the crystal structure. Compound **4** (Pd^II^–Yb^III^–Pd^II^) forms crystals in the space group *C*2/*c*. The coordination unit in **4** is similar to **2** and **3** with one water molecule in the coordination sphere, but in the crystal, instead of three water molecules, there are 5.5 H_2_O.

**Figure 3:**
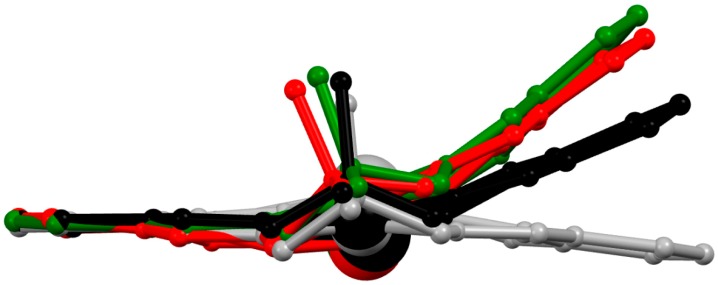
Overlay of selected molecular fragments of coordination units in complexes of **1** (red), **2** (black), **3** (grey) and a previously reported complex of H_4_L with Cu^II^–Nd^III^–Cu^II^ (green) [15]. The superposition was made for the benzene rings.

The preference of a palladium(II) ion to form a planar square polyhedron is sustained in the presented complexes **1**–**4** with a voluminous H_4_L Schiff base ligand. The conformation of the organic ligand has been flattened slightly in comparison to previously reported complexes with Cu^II^–Ln^III^–Cu^II^ (Figure 3) [15]. The valence angles in **1**–**4** between two planes of benzene rings around the palladium(II) ions are in the range of 9–39° (*ψ*, Table 1), whereas the corresponding values for Cu^II^–Ln^III^–Cu^II^ compounds are in the range of 40–47°. The deviation of the Pd^II^ ion from the N_2_O_2_ mean plane is nearly zero (Δ, Table 1). The dihedral angle between the PdO_(phenoxo)2_ and LnO_(phenoxo)2_ planes is also small (in the range of 0–10°, *σ*, Table 1), similar to complexes of H_4_L with Cu^II^ and Ln^III^ metal ions [15] and unlike other 3d-4f complexes. For example, in complexes of Zn^II^–Ln^III^–Zn^II^ with a ligand based on a 2-hydroxypropanyl bridge, the *σ* value was approximately 35° [16]. The dihedral angle between two N_2_O_2_ mean planes in the coordination core in **1**–**4** is in the range of 65–70° (*ε*, Table 1) and the valence angle along the Pd^II^–Ln^III^–Pd^II^ line is in the range of 162–171° (*φ*, Table 1).

### 2.2. Thermal Properties

**Figure 4:**
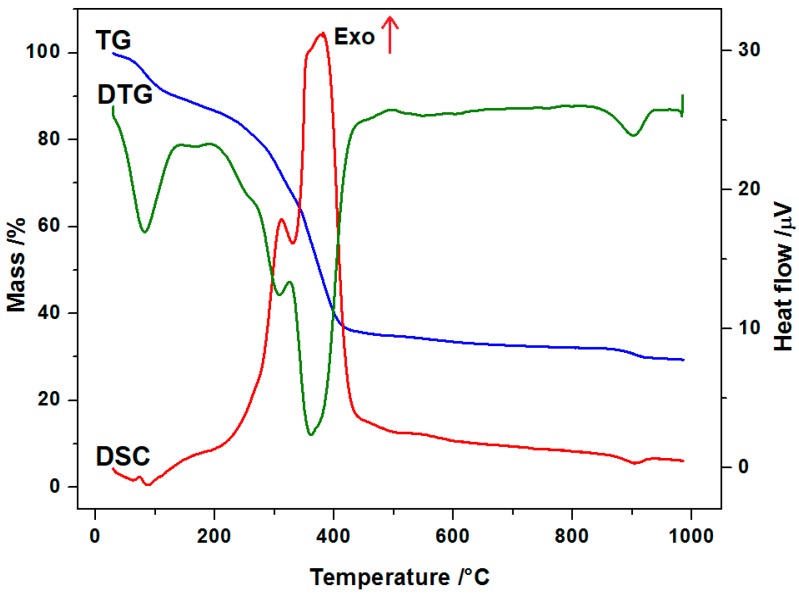
Thermogravimetric (TG), differential thermogravimetric (DTG) and differential scanning calorimetric (DSC) curves of the thermal decomposition of complex Pd^II^–Er^III^–Pd^II^ (**3**) in air (Exo–exoenergetic effect).

In order to examine the thermal behaviour and the stability of the Pd^II^–Ln^III^–Pd^II^ complexes, the thermogravimetric (TG), differential thermogravimetric (DTG) and differential scanning calorimetric (DSC) curves were recorded in air atmosphere. As shown in Figure 4 and Figures S1–S3, the complexes are stable at room temperature.

The recorded TG curves of **1**–**4** display similar mass loss patterns. The samples of compounds during heating up to approximately 110 °C undergo a desolvation process. The desolvation process is accompanied by a small endothermic effect, as seen on the DSC curves. These results are also confirmed by the TG-FTIR analysis. The recorded FTIR spectra of evolved gas phase for the selected complexes (Figure S4) show that water or water and methanol are the main products released during this stage. The characteristic vibration bands in the wavenumber ranges from 4000–3400 cm^−1^ and 2060–1260 cm^−1^ corresponding to the stretching and deforming vibrations of H_2_O molecules, whereas the bands associated with the presence of CH_3_OH are observed at 3368 cm^−1^, 1307 cm^−1^ and 1095 cm^−1^, respectively [17]. The decomposition process of the Pd^II^–Ln^III^–Pd^II^ complexes is intricate and it was not possible to distinguish intermediate solid products. The final products (mixtures of: PdO, Pd and respective lanthanide oxides Eu_2_O_3_, Tb_2_O_3_, Tb_4_O_7_, Er_2_O_3_, Yb_2_O_3_) obtained during thermal decomposition in air were experimentally verified by X-ray diffraction powder patterns (Figures S5–S8) [18,19,20].

### 2.3. Absorption and Luminescence Spectra

In order to determine the photophysical properties of the obtained complexes, UV-Vis excitation and emission spectra were measured.

The absorption spectra of the Schiff base ligand (H_4_L) and the Pd^II^–Ln^III^–Pd^II^ complexes dissolved in methanol are shown in Figure S9 (Supplementary Material), and the absorption data are summarized in Table 2. The H_4_L ligand shows the two most intense absorption bands, with maxima centered at 218 nm and 264.5 nm. All transitions correspond to the π-π* and n-π* transitions in the ligand [16,21]. The absorption spectra of the synthesized Pd^II^–Ln^III^–Pd^II^ complexes with H_4_L are characterized by two absorption maxima in the ultraviolet region between 210–290 nm and an additional small band centered at approximately 375 nm, as shown in Figure S9. The higher-energy bands are attributable to the intra-ligand transitions while the small bands could be assigned to metal-to-ligand charge transfer or a mixture of metal-to-ligand charge transfer and ligand-centered transitions [22,23,24]. No absorption bands due to f-f transitions were observed in the absorption spectra of these compounds due to the fact that the f-f transitions in Ln^III^ ions are very weak [25]. As a consequence of complex formation, bathochromic shifts are observed in the absorption spectra of solutions of the Pd^II^–Ln^III^–Pd^II^ complexes.

**Figure 5:**
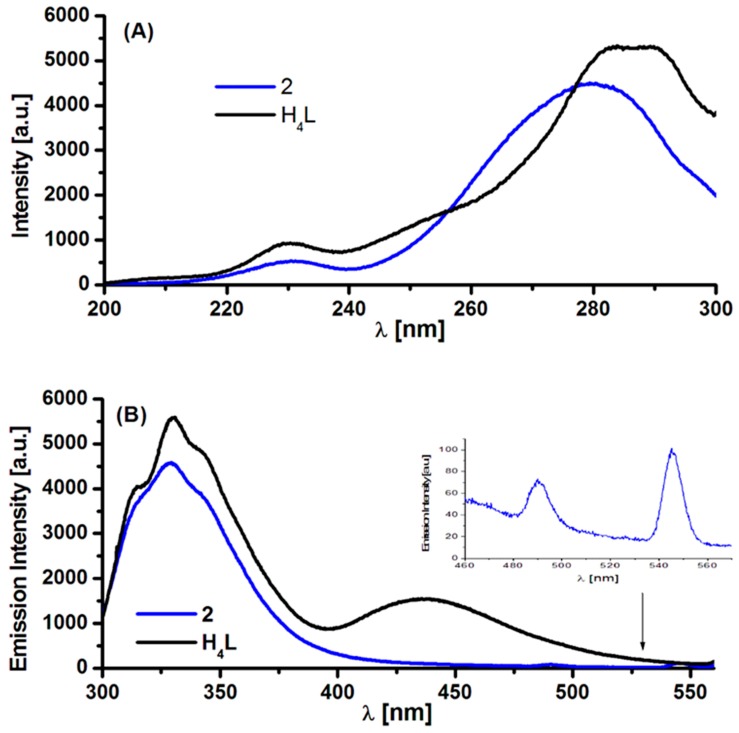
The excitation (**A**) and emission (**B**) spectra of methanolic solutions of H_4_L ligand and complex **2**, the inset presents the f-f transitions in the Tb^III^ ion.

The luminescence and excitation spectra of the Schiff base ligand and its corresponding Pd^II^–Ln^III^–Pd^II^ complexes dissolved in methanol and in the solid state were measured at room temperature. As shown in Figure 5A, the solution of the free H_4_L ligand exhibits unsymmetrical emission with two bands at 330.6 nm and 438.2 nm upon photoexcitation at λ_ex_ = 283 nm. These bands can be assigned to intra-ligand transitions. In the case of the methanolic solutions of complexes, upon excitation at approximately λ_ex_ = 280 nm, the emissions at λ = 330 nm are slightly weaker than the corresponding emission in the ligand and, in addition, the blue emission band present in the ligand disappears (Figure S10). Complex **2** (Figure 5B) shows two very weak Tb^3+^ luminescence bands, corresponding to ^5^D_4_-^7^F_6_ (490.0 nm) and ^5^D_4_-^7^F_5_ (545.6 nm) transitions. The spectra of the other complexes are devoid of any peaks characteristic of metal-centered luminescence. The results of our luminescence experiments show that the low efficiency of the process of intramolecular energy transfer takes place only in complex **2**.

The excitation and emission spectra of the ligand and Pd^II^–Ln^III^–Pd^II^ complexes in solid state are shown in Figures S11 and S12, respectively. The maximum luminescence intensities in the excitation and emission spectra of microcrystalline H_4_L ligand are observed at wavelengths of 380 nm, 418.0 nm and 573 nm (Table 2). As can be seen in Figure S12, the corresponding emission spectra of all reported complexes show weak emission bands at about 469 nm and 494 nm when excited at approximately 380 nm. The emission bands are bathochromic shifted compared to the corresponding ligand (λ_em_ = 418 nm). In these systems, emissions probably originate from the metal to ligand charge transfer (MLCT) state with a mix of intra-ligand transfer [26]. Furthermore, in these emission spectra, the luminescence intensity of the ligand dramatically decreases after complex formation with palladium and lanthanide ions and its yellow emission completely disappears (Figure S12).

## 3. Materials and Methods

### 3.1. Materials

All the reagents: 2,3-dihydroxybenzaldehyde, 2,2-dimethyl-1,3-propanediamine, bis(benzonitrile)palladium(II) chloride, Eu(NO_3_)_3_·5H_2_O, Tb(NO_3_)_3_·5H_2_O, Er(NO_3_)_3_·5H_2_O, Yb(NO_3_)_3_·5H_2_O and CH_3_OH (solvent) were purchased from commercial sources and were used without further purification.

### 3.2. Synthesis of the **H_4_L**

*N*,*N*′-bis(2,3-dihydroxybenzylidene)-1,3-diamino-2,2-dimethylpropane (abbreviated as H_4_L) was prepared following a literature procedure [27,28]. The crystal structure was reported previously [15]. 2,2-Dimethyl-1,3-propanediamine (0.51 g, 5 mmol, 5 mL methanol) was added dropwise to 2,3-dihydroxybenzaldehyde (1.38 g, 10 mmol) dissolved in hot methanol (50 mL). The obtained compound was separated as orange needles and recrystallized twice from methanol. The empirical formula and the molecular weight are C_19_H_22_N_2_O_4_ and 342.00 g/mol respectively. Yield 85%. Analytical data (%), calculated: C, 66.60; H, 6.43; N, 8.19. Found: C, 66.40; H, 6.20; N, 8.15. IR (ATR); 3194 m, 1624 vs, 1547 m, 1515 m, 1463 m, 1405 m, 1382 m, 1349 m, 1234 s, 1181 vs, 1078 w, 1053 w, 1022 s, 895 w, 849 c, 781 w, 734 vs, 644 w. ^1^H NMR (500 MHz, CDCl_3_, δ ppm): 1.13 (s, 6H, CH3), 3.52 (s, 4H, CH2), 6.72 (t, *J* = 7.9 Hz, 2H, CH), 6.81 (dd, *J* = 7.9, 1.3 Hz, 2H, CH), 6.99 (dd, *J* = 7.9, 1.3 Hz, 2H, CH), 8.26 (s, 2H, CH), 14.00 (bs, 2H, OH). ^13^C NMR (126 MHz, DMSO-*d*_6_, δ ppm): 23.95, 36.19, 66.43, 118.13, 118.21, 118.51, 122.32, 146.35, 151.84, 167.37.

### 3.3. General Procedure of Preparation of Pd^II^–Ln^III^–Pd^II^ Complexes

The heterotrinuclear complexes have been synthesized using the following general procedure: H_4_L (0.1368 g, 0.4 mmol) was dissolved in methanol (20 mL). Next, Pd(C_6_H_5_CN)_2_Cl_2_ (0.1534 g, 0.4 mmol) was added to this solution. The reaction mixture was refluxed for 0.5 h. At this stage, Eu(NO_3_)_3_·5H_2_O (0.2 mmol, 0.0856 g), Tb(NO_3_)_3_·5H_2_O (0.2 mmol, 0.0870 g), Er(NO_3_)_3_·5H_2_O (0.2 mmol, 0.0887 g) or Yb(NO_3_)_3_·5H_2_O (0.2 mmol, 0.0898 g) dissolved in methanol (5 mL), was added and the reaction mixture was stirred at about 45 °C for a further period of 0.5 h to afford a clear solution. The pale brown-coloured solution was kept in a refrigerator for crystallization. After about a week, crystals suitable for X-ray crystallography were obtained. The characterization data for these complexes are given below.

#### 3.3.1. [Pd_2_Eu(H_2_L)_2_NO_3_](NO_3_)_2_∙2H_2_O∙2CH_3_OH (**1**)

The empirical formula and the molecular weight are C_40_H_52_N_7_O_21_Pd_2_Eu and 1331.64 g/mol, respectively. Yield 29 %. Analytical data (%), calculated: C, 36.08; H, 3.94; N, 7.36; Pd, 15.98; Eu, 11.41. Found: C, 36.20; H, 3.69; N, 7.40; Pd, 16.10; Eu, 11.50. IR (ATR); 3200 w, 1646 vs, 1568 w, 1464 s, 1404 w, 1388 w, 1302 m, 1256 vs, 1219 s, 1170 w, 1097 w, 1052 w, 871 m, 780 m, 759 w, 732 s, 675 s, 552 w.

#### 3.3.2. [Pd_2_Tb(H_2_L)2H_2_O](NO_3_)_3_∙3H_2_O (**2**)

The empirical formula and the molecular weight are C_38_H_48_N_7_O_21_Pd_2_Tb and 1310.55 g/mol, respectively. Yield 30%. Analytical data (%), calculated: C, 34.82; H, 3.69; N, 7.48; Pd, 16.24; Tb, 12.13. Found: C, 34.60; H, 3.50; N, 7.30; Pd, 16.00; Tb, 12.20. IR (ATR); 3218 w, 1647 vs, 1568 w, 1464 m, 1388 w, 1308 m, 1256 s, 1219 s, 1170 w, 1097 w, 1040 w, 869 m, 816 w, 780 m, 753 w, 732 vs, 656 s, 552 w.

#### 3.3.3. [Pd_2_Er(H_2_L)_2_H_2_O](NO_3_)_3_∙3H_2_O (**3**)

The empirical formula and the molecular weight are C_38_H_48_N_7_O_21_Pd_2_Er and 1318.92 g/mol, respectively. Yield 32%. Analytical data (%), calculated: C, 34.60; H, 3.67; N, 7.43; Pd, 16.14; Er, 12.68. Found: C, 34.50; H, 3.70; N, 7.30; Pd, 16.00; Er, 12.50. IR (ATR); 3204 w, 2959 w, 1646 vs, 1606 vs, 1567 w, 1464 s, 1404 w, 1388 w, 1301 m, 1256 vs, 1219 s, 1170 w, 1097 w, 1052 w, 871 m, 780 m, 759 w, 731 s, 675 s, 552 w.

#### 3.3.4. [Pd_2_Yb(H_2_L)_2_H_2_O](NO_3_)_3_∙5.5H_2_O (**4**)

The empirical formula and the molecular weight are C_38_H_53_N_7_O_23.5_Pd_2_Yb and 1369.75 g/mol, respectively. Yield 32%. Analytical data (%), calculated: C, 33.32; H, 3.90; N, 7.16; Pd, 15.54; Yb, 12.63. Found: C, 33.20; H, 3.70; N, 7.30; Pd, 15.30; Yb, 12.40. IR (ATR); 3201 w, 2964 w, 1655 w, 1607 vs, 1568 w, 1463 s, 1386 w, 1302 m, 1254 s, 1218 s, 1172 w, 1097 w, 1052 w, 871 m, 780 m, 759 w, 733 s, 680 m, 658 m, 555 w.

### 3.4. Methods

The contents of carbon, hydrogen and nitrogen in the complexes were determined by elemental analysis using a CHN 2400 Perkin Elmer analyser. The contents of palladium and lanthanides were established using ED XRF spectrophotometer (Canberra-Packard). The infrared spectra were acquired using a Thermo Scientific Nicolet 6700 FTIR with a Smart iTR diamond ATR accessory. Data were collected between 4000 cm^−1^ and 520 cm^−1^, with a resolution of 4 cm^−1^ for 16 scans. The NMR spectra were recorded on Bruker Avance 500MHz in CDCl_3_ and DMSO-*d*_6_ solutions. Thermal analyses of complexes were carried out by the thermogravimetric (TG) and differential scanning calorimetry (DSC) methods using the SETSYS 16/18 analyser (Setaram). The experiments were carried out under air flow in the temperature range of 20–1000 °C at a heating rate of 10 °C·min^−1^. The samples 5.65 mg (**1**), 7.14 mg (**2**), 5.76 mg (**3**), 7.01 mg (**4**), 6.76 mg (**5**) and 6.02 mg (**6**) were heated in Al_2_O_3_ crucibles. The TG–FTIR of the selected compounds was recorded using the TGA Q5000 analyser (TA Instruments, New Castle, DE, USA), interfaced to the Nicolet 6700 FTIR spectrophotometer (Thermo Scientific). The complex samples were put in an open platinum crucible and heated from ambient temperature to 700 °C. The analysis was carried out at a heating rate of 20 °C min^−1^ under nitrogen at a flow rate of 20 mL min^−1^. To reduce the possibility of gasses condensing along the transfer line, the temperature in the gas cell and transfer line was set to 250 °C and 240 °C, respectively. Gas analysis was performed by matching the spectra against those from the spectrum library (Nicolet TGA Vapor Phase) of the software Ominic, together with the literature sources. The X-ray powder diffraction patterns of the products of the decomposition process were collected at room temperature on an Empyrean PANanalytical automated powder diffractometer with CuKα radiation (λ = 1.54187 Å) over the scattering angular range 2*θ* = 20–90°.

The electronic absorption spectra of the ligand H_4_L and complexes **1**–**4** were recorded with a Shimadzu UV 2401PC spectrophotometer, in 10 × 10 mm quartz cells, in methanol solutions at a concentration of about 2 × 10^−5^ mol/L. Excitation and emission spectra, in methanol solution and in solid state, were measured at ambient temperature and for the UV-Vis range, a Hitachi 7000 Fluorescence Spectrophotometer was used. The solid state samples were measured within the same sample holder to ensure a consistent amount of luminescent materials in all samples. Excitation and emission spectra were corrected for the instrumental response. All measurements were carried out under the same experimental conditions.

Diffraction data for **1**–**4** were collected at room temperature on a SuperNova X-ray diffractometer equipped with an Atlas S2 CCD detector using mirror-monochromatized CuK*α* radiation (λ = 1.54184 Å). Absorption effects were corrected with CrysAlisPro 1.171.38.46 [29] by empirical absorption correction using spherical harmonics, implemented in the SCALE3 ABSPACK scaling algorithm. The structures were solved by direct methods using the ShelXT [30] structure solution program using intrinsic phasing and refined with the Olex2.refine refinement package using Gauss-Newton minimisation [31]. For nitrate ions in **1** and **2**, the bond length restraints were applied by DFIX instructions to 1.21(1) and DANG 2.10(2) Å for N–O and O–O distances, respectively. Non-hydrogen atoms, except of nitrate N and O atoms and solvent molecules, were refined with anisotropic displacement parameters. The C-bound H atoms were positioned geometrically and the ‘riding’ model for the C–H bonds was used in the refinement. The structure **4** contains solvent accessible voids of 118 Å^3^, which can be attributed to disordered solvent molecules (32 electrons). The summary of the experimental details and the crystal structure refinement parameters are given in Table 1 and Table S1. The experimental details and final atomic parameters have been deposited with the Cambridge Crystallographic Data Centre as supplementary material (CCDC: 1865659-1865662). The data can be obtained free of charge from The Cambridge Crystallographic Data Centre via www.ccdc.cam.ac.uk/structures.

## 4. Conclusions

Four novel 4d-4f Schiff base coordination compounds of palladium(II) and lanthanides(III) have been synthesized and characterized by X-ray diffraction structural studies, thermal analysis, and by luminescence measurements. The analysis of the geometry of the unique crystal structures **1**–**4** extended the knowledge about specific requirements necessary for the design and synthesis of new heterometallic Pd^II^–Ln^III^ coordination compounds. A planar square coordination site for Pd^II^ ions is beneficial. The presented results show that such geometry is also available for voluminous two-compartmental Schiff base ligands.

The complexes **1**–**4** are stable at room temperature. Their desolvation process is consistent with the loss of water and methanol molecules in the case of **1**, or of water in **2**–**4**. The main differences originate from the kind and amount of solvent molecules outside the coordination sphere and the type of small ligands coordinated to lanthanide(III) ions (nitrate ion (**1**) or water molecule (**2**–**4**)).

The photoluminescent properties were measured at ambient temperature, revealing similar emission patterns. The **1**–**4** complexes have been shown to be poorly effective emitters. The quenching of yellow emission of the *N*,*N*′-bis(2,3-dihydroxybenzylidene)-1,3-diamino-2,2-dimethylpropane upon complexation by palladium(II) and lanthanide(III) ions may be used for analytical applications to detect metal ions. Further studies are required.

## Figures and Tables

**Table 1:** Selected structural parameters^a^ for complexes **1**–**4**.

Parameter	1	2	3	4
Ln1–Pd1	3.612(3)	3.5776(18)	3.529(2)	3.5267(7)
Ln1–Pd2	3.618(3)	3.5615(18)	3.546(2)	3.5411(7)
Ln1–O1	2.283(19)	2.348(16)	2.348(16)	2.362(6)
Ln1–O2	2.482(17)	2.290(15)	2.290(15)	2.308(6)
Ln1–O3	2.532(19)	2.470(18)	2.470(18)	2.451(6)
Ln1–O4	2.635(19)	2.376(18)	2.376(18)	2.385(6)
Ln1–O5	2.396(19)	2.337(13)	2.337(13)	2.334(6)
Ln1–O6	2.46(2)	2.349(16)	2.349(16)	2.341(6)
Ln1–O7	2.49(2)	2.425(17)	2.425(17)	2.428(6)
Ln1–O8	2.40(3)	2.552(18)	2.552(18)	2.394(7)
Ln1–O9	2.57(3)	2.43(2)	2.43(2)	2.328(7)
Ln1–O10	2.56(2)	–	–	–
Pd1–O1	2.070(15)	2.019(15)	1.979(13)	1.980(6)
Pd1–O2	1.954(16)	2.044(13)	1.994(13)	1.990(6)
Pd1–N1	1.914(18)	1.96(2)	1.949(14)	1.999(7)
Pd1–N2	2.07(2)	2.039(18)	1.985(17)	1.991(7)
Pd2–O5	1.989(17)	1.956(14)	2.008(14)	1.989(6)
Pd2–O6	1.99(3)	1.998(13)	1.991(13)	1.984(6)
Pd2–N3	2.00(2)	2.018(19)	1.989(16)	1.988(8)
Pd2–N4	1.906(19)	1.95(2)	2.006(16)	1.995(8)
Pd1–O1–Ln1	112.1(7)	109.8(9)	110.8(9)	108.3(2)
Pd1–O2–Ln1	108.5(7)	111.1(8)	111.6(7)	110.1(2)
Pd2–O5–Ln1	110.9(8)	111.8(8)	109.7(8)	109.7(3)
Pd2–O6–Ln1	108.4(10)	109.8(8)	111.1(8)	109.6(3)
Δ*^b^* (Pd1; Pd2)	0.02; 0.01	0.02; 0.02	0.03; 0	0.02; 0
*σ^c^* (Pd1; Pd2)	10.5; 2.1	0.7; 9.2	3.0; 8.5	3.6; 4.4
*ε^d^*	65.34	67.62	70.40	68.02
*φ^e^*	161.70(8)	170.26(6)	170.92(6)	169.45(2)
*ψ^f^* (Pd1; Pd2)	29.2; 39.2	25.2; 10.3	25.7; 11.6	27.6; 9.43

^a^ Distances in Å, angles in degrees, Ln = Eu (**1**), Tb (**2**), Er (**3**), Yb (**4**), respectively; Δ*^b^*—deviation of Pd^II^ ion from the N_2_O_2_ mean plane; *σ^c^*—dihedral angle between PdO_(phenoxo)2_ and LnO_(phenoxo)2_ planes; *ε^d^—*dihedral angle between two N_2_O_2_ mean planes in the coordination core; *φ^e^*—valence angle along the Pd^II^–Ln^III^–Pd^II^ line; *ψ^f^*—dihedral angle between two planes of benzene rings around the Pd^II^ ion.

**Table 2:** Photophysical parameters of studied complexes **1**–**4** and H_4_L ligand (in nm).

Compound	λmax (abs) ^(a)^	λex/λem ^(a)^	λex/λem ^(b)^
**H_4_L**	218.0; 264.5; 298.5; 424.0	283/330.6; 438.2	380/418.0; 573.0
**1**	214.0; 286.0	280/329.4	381/465.4; 496.2
**2**	215.0; 285.5	280/329.2	380/469.6; 492.4
**3**	213.0; 284.0	281/328.6	381/469.0; 494.2
**4**	216.0; 286.0	279/327.8	382/472.2; 496.1

^(a)^ in methanol solution, ^(b)^ in solid state.

## Supplementary Materials

The following materials are available online, Table S1. Crystallographic data for complexes **1**–**4**, Figure S1. TG, DTG, and DSC curves of Pd^II^–Eu^III^–Pd^II^ (**1**) in air, Figure S2. TG, DTG and DSC curves of Pd^II^–Tb^III^–Pd^II^ (**2**) in air, Figure S3. TG, DTG and DSC curves of Pd^II^–Yb^III^–Pd^II^ (**4**) in air, Figure S4. FTIR spectra of gaseous products of the Pd^II^–Eu^III^–Pd^II^ (**1**) decomposition, Figure S5. The X-ray powder diffraction patterns of the final products of Pd^II^–Eu^III^–Pd^II^ (**1**) decomposition in air, Figure S6. The X-ray powder diffraction patterns of the final products of Pd^II^–Tb^III^–Pd^II^ (**2**) decomposition in air, Figure S7. The X-ray powder diffraction patterns of the final products of Pd^II^–Er^III^–Pd^II^ (**3**) decomposition in air, Figure S8. The X-ray powder diffraction patterns of the final products of Pd^II^–Yb^III^–Pd^II^ (**4**) decomposition in air, Figure S9. Absorption spectra of Schiff base ligand H_4_L and its metal-mixed complexes **1**–**4** dissolved in methanol (c~2·10^−5^M), Figure S10. Excitation (A) and emission (B) spectra of methanolic solutions of reported complexes, Figure S11. Luminescence spectrum of ligand H_4_L in solid state; the inset is the excitation spectrum, Figure S12. Luminescence spectra of reported complexes in solid state, the inset present the excitation spectra of selected complexes, Figure S13. ^1^H NMR spectrum of ligand H_4_L in CDCl_3_ solution, Figure S14. ^13^C NMR spectrum of ligand H_4_L in DMSO-*d*_6_ solution.

## References

[1] Manabe K. (2015). Palladium Catalysts for Cross-Coupling Reaction. Catalysts.

[2] Roy D., Uozumi Y. (2018). Recent Advances in Palladium-Catalyzed Cross-Coupling Reactions at Ppm to Ppb Molar Catalyst Loadings. Adv. Synth. Catal..

[3] Szwaczko K., Demchuk O.M., Mirosław B., Strzelecka D., Pietrusiewicz K.M. (2016). Straightforward Approach to Norbornene Core Based Chiral Ligands by Tandem Cross Dehydrogenative Coupling Reactions. Tetrahedron Lett..

[4] Albeniz A.C., Espinet P. (2006). Palladium: Inorganic & Coordination Chemistry. Encyclopedia of Inorganic Chemistry.

[5] Balamurugan R., Liu J.-H., Liu B.T. (2018). A Review of Recent Developments in Fluorescent Sensors for the Selective Detection of Palladium Ions. Coord. Chem. Rev..

[6] Groom C.R., Bruno I.J., Lightfoot M.P., Ward S.C. (2016). The Cambridge Structural Database. Acta Cryst. Sect. B Struct. Sci. Cryst. Eng. Mater..

[7] Watanabe R., Fujiwara K., Okazawa A., Tanaka G., Yoshii S., Nojiri H., Ishida T. (2011). Chemical Trend of Ln–M Exchange Couplings in Heterometallic Complexes with Ln = Gd, Tb, Dy, Ho, Er and M = Cu, V. Chem. Commun..

[8] Pasatoiu T.D., Tiseanu C., Madalan A.M., Jurca B., Duhayon C., Sutter J.P., Andruh M. (2011). Study of the Luminescent and Magnetic Properties of a Series of Heterodinuclear [Zn ^II^ Ln ^III^ ] Complexes. Inorg. Chem..

[9] Andruh M., Costes J.-P., Diaz C., Gao S. (2009). 3d-4f Combined Chemistry: Synthetic Strategies and Magnetic Properties. Inorg. Chem..

[10] Yang X.-P., Jones R.A., Wong W.-K., Lynch V., Oye M.M., Holmes A.L. (2006). Design and Synthesis of a near Infra-Red Luminescent Hexanuclear Zn–Nd Prism. Chem. Commun..

[11] Li B., Huang R.-W., Zang S.-Q., Mak T.C.W. (2013). Assembly of Silver(i)–organic Frameworks from Flexible Supramolecular Synthons with Pendant Ethynide Arm Attached to Biphenyl and Phenoxybenzene Skeletons. CrystEngComm.

[12] Amirkhanov O.V., Moroz O.V., Znovjyak K.O., Sliva T.Y., Penkova L.V., Yushchenko T., Szyrwiel L., Konovalova I.S., Dyakonenko V.V., Shishkin O.V. (2014). Heterobinuclear Zn-Ln and Ni-Ln Complexes with Schiff-Base and Carbacylamidophosphate Ligands: Synthesis, Crystal Structures, and Catalytic Activity. Eur. J. Inorg. Chem..

[13] Sreejith S.S., Mohan N., Aiswarya N., Kurup M.R.P. (2016). Inclusion, Pseudo-Inclusion Compounds and Coordination Polymer of Pd(II), Zn(II) and Cd(II) from Salen-Type Schiff Base Ligand with a 1,3-Diimino Spacer Group: Crystal Structures, Spectroscopic and Thermal Studies. Polyhedron.

[14] Zhang G. (2012). A Heterotrimetallic Pd–Sm–Pd Complex for Asymmetric Friedel–Crafts Alkylations of Pyrroles with Nitroalkenes. Org. Biomol. Chem..

[15] Cristóvão B., Osypiuk D., Miroslaw B., Bartyzel A. (2018). Syntheses, Crystal Structures, Thermal and Magnetic Properties of New Heterotrinuclear Cu^II^–Ln^III^–Cu^II^ complexes Incorporating N_2_O_4_-Donor Schiff Base Ligands. Polyhedron.

[16] Miroslaw B., Cristóvão B., Hnatejko Z., Miroslaw B., Cristóvão B., Hnatejko Z. (2018). Heterometallic Zn^II^–Ln^III^–Zn^II^ Schiff Base Complexes with Linear or Bent Conformation—Synthesis, Crystal Structures, Luminescent and Magnetic Characterization. Molecules.

[17] Bartyzel A. (2013). Synthesis, Crystal Structure and Characterization of manganese(III) Complex Containing a Tetradentate Schiff Base. J. Coord. Chem..

[18] Guney E., Yilmaz V.T., Buyukgungor O. (2011). Palladium(II) and platinum(II) Saccharinate Complexes Containing Pyridine and 3-Acetylpyridine: Synthesis, Crystal Structures, Fluorescence and Thermal Properties. Polyhedron.

[19] Guney E., Yilmaz V.T., Buyukgungor O. (2010). Neutral and Cationic palladium(II) and platinum(II) Complexes of 2,2′-Dipyridylamine with Saccharinate: Syntheses, Spectroscopic, Structural, Fluorescent and Thermal Studies. Inorg. Chim. Acta.

[20] Guney E., Yilmaz V.T., Kazak C. (2010). Bis(saccharinato)palladium(II) and platinum(II) Complexes with 2,2′-Bipyridine: Syntheses, Structures, Spectroscopic, Fluorescent and Thermal Properties. Polyhedron.

[21] Zhang D., Gao B., Li Y. (2017). Synthesis and Luminescence Properties of Polymer-Rare Earth Complexes Containing Salicylaldehyde-Type Bidentate Schiff Base Ligand. Luminescence.

[22] Mandal M., List M., Teasdale I., Redhammer G., Chakraborty D., Monkowius U. (2018). Palladium Complexes Containing Imino Phenoxide Ligands: Synthesis, Luminescence, and Their Use as Catalysts for the Ring-Opening Polymerization of Rac-Lactide. Monatsh. Chem..

[23] Seyfi S., Alizadeh R., Ganji M.D., Amani V. (2017). Palladium(II) Complexes with 1,2,4-Triazole Derivative; Ethylene Diamine as Ligands, Synthesis, Characterization, Luminesence Study; Crystal Structure Determination. Polyhedron.

[24] Micutz M., Iliş M., Staicu T., Dumitraşcu F., Pasuk I., Molard Y., Roisnel T., Cîrcu V. (2014). Luminescent Liquid Crystalline Materials Based on Palladium(II) Imine Derivatives Containing the 2-Phenylpyridine Core. Dalt. Trans..

[25] Kaczmarek M.T., Kubicki M., Hnatejko Z. (2015). Two Types of Lanthanide Schiff Base Complexes: Synthesis, Structure and Spectroscopic Studies. Polyhedron.

[26] Ivanov M.A., Puzyk M.V. (2001). Spectral and Luminescent Specific Features of Phenylpyridine Ethylenediamine Complexes of Pt(II), Pd(II), and Au(III). Opt. Spectrosc..

[27] Aguiari A., Bullita E., Casellato U., Guerriero P., Tamburini S., Vigato P.A. (1992). Macrocyclic and Macroacyclic Compartmental Schiff Bases: Synthesis, Characterization, X-Ray Structure and Interaction with Metal Ions. Inorg. Chim. Acta.

[28] Bermejo M.R., Fernández M.I., Gómez-Fórneas E., González-Noya A., Maneiro M., Pedrido R., Rodríguez M.J. (2007). Self-Assembly of Dimeric Mn^III^–Schiff-Base Complexes Tuned by Perchlorate Anions. Eur. J. Inorg. Chem..

[29] (2016). Crysalis-Pro Software System.

[30] Sheldrick G.M. (2015). SHELXT—Ntegrated Space-Group and Crystal-Structure Determination. Acta Crystallogr. Sect. A Found. Crystallogr..

[31] Dolomanov O.V., Bourhis L.J., Gildea R.J., Howard J.A.K., Puschmann H. (2009). OLEX2: A Complete Structure Solution, Refinement and Analysis Program. J. Appl. Crystallogr..

