# Supplementary material for: Structural, Luminescent and Thermal Properties of Heteronuclear PdII–LnIII–PdII Complexes of Hexadentate N2O4 Schiff Base Ligand
Source: Molecules. 2018 Sep 21;23(10):2423. doi: 10.3390/molecules23102423 (PMC6222701; doi:10.3390/molecules23102423)
Supplement: Supplementary file 1 [file molecules-23-02423-s001.zip › Supplementary Materials.pdf]

Molecules

Structural, luminescent and thermal properties of heteronuclear Pd^{II}–Ln^{III}–Pd^{II} complexes of hexadentate N_2O_4 Schiff base

Barbara Miroslaw [1,*], Beata Cristóvão [2] and Zbigniew Hnatejko [3]

[1] Department of Crystallography, Maria Curie-Sklodowska University, Maria Curie-Sklodowska sq. 3, 20-031 Lublin, Poland
[2] Department of General and Coordination Chemistry, Maria Curie-Sklodowska University, Maria Curie-Sklodowska sq. 2, 20-031 Lublin, Poland; beata.cristovao@poczta.umcs.lublin.pl
[3] Department of Rare Earths, Faculty of Chemistry, Adam Mickiewicz University in Poznań, Umultowska 89b, 61-614 Poznań, Poland; zbychuh@amu.edu.pl
* Correspondence: barbara.miroslaw@umcs.lublin.pl; Tel.: +48 81 537 55 82

List of items:

- **Table S1.** Crystallographic data for complexes **1-4.**
- **Figure S1.** TG, DTG, and DSC curves of Pd^{II}–Eu^{III}–Pd^{II} (**1**) in air.
- **Figure S2.** TG, DTG and DSC curves of Pd^{II}–Tb^{III}–Pd^{II} (**2**) in air.
- **Figure S3.** TG, DTG and DSC curves of Pd^{II}–Yb^{III}–Pd^{II} (**4**) in air.
- **Figure S4.** FTIR spectra of gaseous products of the Pd^{II}-Eu^{III}-Pd^{II} (**1**) decomposition.
- **Figure S5.** The X-ray powder diffraction patterns of the final products of Pd^{II}–Eu^{III}–Pd^{II} (**1**) decomposition in air.
- **Figure S6.** The X-ray powder diffraction patterns of the final products of Pd^{II}–Tb^{III}–Pd^{II} (**2**) decomposition in air.
- **Figure S7.** The X-ray powder diffraction patterns of the final products of Pd^{II}–Er^{III}–Pd^{II} (**3**) decomposition in air.
- **Figure S8.** The X-ray powder diffraction patterns of the final products of Pd^{II}–Yb^{III}–Pd^{II} (**4**) decomposition in air.
- **Figure S9.** Absorption spectra of Schiff base ligand **H_4L** and its metal-mixed complexes **1-4** dissolved in methanol ($c \sim 2 \cdot 10^{-5}$M).
- **Figure S10.** Excitation (A) and emission (B) spectra of methanolic solutions of reported complexes.
- **Figure S11.** Luminescence spectrum of ligand **H_4L** in solid state; the inset is the excitation spectrum.
- **Figure S12.** Luminescence spectra of reported complexes in solid state, the inset present the excitation spectra of selected complexes.
- **Figure S13.** ^{1}H NMR spectrum of ligand **H_4L** in $CDCl_3$ solution.
- **Figure S14.** ^{13}C NMR spectrum of ligand **H_4L** in DMSO-d6 solution.

Table S1. Crystallographic data for complexes **1**-**4**.

**Identification code**	**1**	**2**	**3**	**4**
Empirical formula	$C_{40}H_{52}N_7O_{21}Pd_2Eu$	$C_{38}H_{48}N_7O_{21}Pd_2Tb$	$C_{38}H_{48}N_7O_{21}Pd_2Er$	$C_{38}H_{53}N_7O_{23.5}Pd_2Yb$
Formula weight	1331.64	1310.55	1318.93	1369.71
Temperature/K	295.1(4)	294.7(5)	294.2(5)	294.6(5)
Crystal system	monoclinic	monoclinic	monoclinic	monoclinic
Space group	$P2_1/c$	$P2_1/n$	$P2_1/n$	$C2/c$
a/Å	13.7939(9)	16.3535(7)	16.3366(10)	27.3457(6)
b/Å	20.1474(16)	17.3370(14)	17.1526(16)	18.2653(4)
c/Å	18.218(2)	17.2109(10)	17.2567(13)	21.4206(5)
β/°	91.830(7)	103.233(5)	104.035(8)	101.203(2)
Volume/Å^3	5060.3(8)	4750.1(5)	4691.2(7)	10495.2(4)
Z	4	4	4	8
ρ_{calc}/ g/cm^3	1.748	1.833	1.8673	1.734
μ/mm^{-1}	15.147	13.991	10.072	9.391
F(000)	2656.0	2600.0	2584.0	5440.0
Crystal size/mm^3	0.2 × 0.15 × 0.15	0.25 × 0.1 × 0.01	0.2 × 0.05 × 0.03	0.35 × 0.1 × 0.03
Radiation	CuKα (λ = 1.54184)	CuKα (λ = 1.54184)	Cu Kα (λ = 1.54184)	CuKα (λ = 1.54184)
2Θ range /°	7.77 to 135.362	6.728 to 135.352	7.38 to 135.36	6.59 to 135.366
Reflections collected	32903	30420	30339	36265
Independent reflections	9119 [R_{int} = 0.1160, R_{sigma} = 0.0928]	8600 [R_{int} = 0.1061, R_{sigma} = 0.0905]	8476 [R_{int} = 0.0983, R_{sigma} = 0.1147]	9504 [R_{int} = 0.0347, R_{sigma} = 0.0299]
Data/restr./parameters	9119/18/638	8600/18/597	8476/17/626	9504/24/627
Goodness-of-fit on F^2	1.238	1.177	1.094	1.050
Final R indexes [I>=2σ (I)]	R_1 = 0.1518, wR_2 = 0.3886	R_1 = 0.1355, wR_2 = 0.3292	R_1 = 0.1291, wR_2 = 0.1885	R_1 = 0.0670, wR_2 = 0.2081
Final R indexes [all data]	R_1 = 0.2401, wR_2 = 0.4722	R_1 = 0.2062, wR_2 = 0.4140	R_1 = 0.1871, wR_2 = 0.2370	R_1 = 0.0798, wR_2 = 0.2336
Larg. diff. peak/hole / e Å^{-3}	1.98/-1.17	1.03/-2.95	3.30/-2.84	3.86/-2.33
CCDC No.	1865659	1865660	1865662	1865661

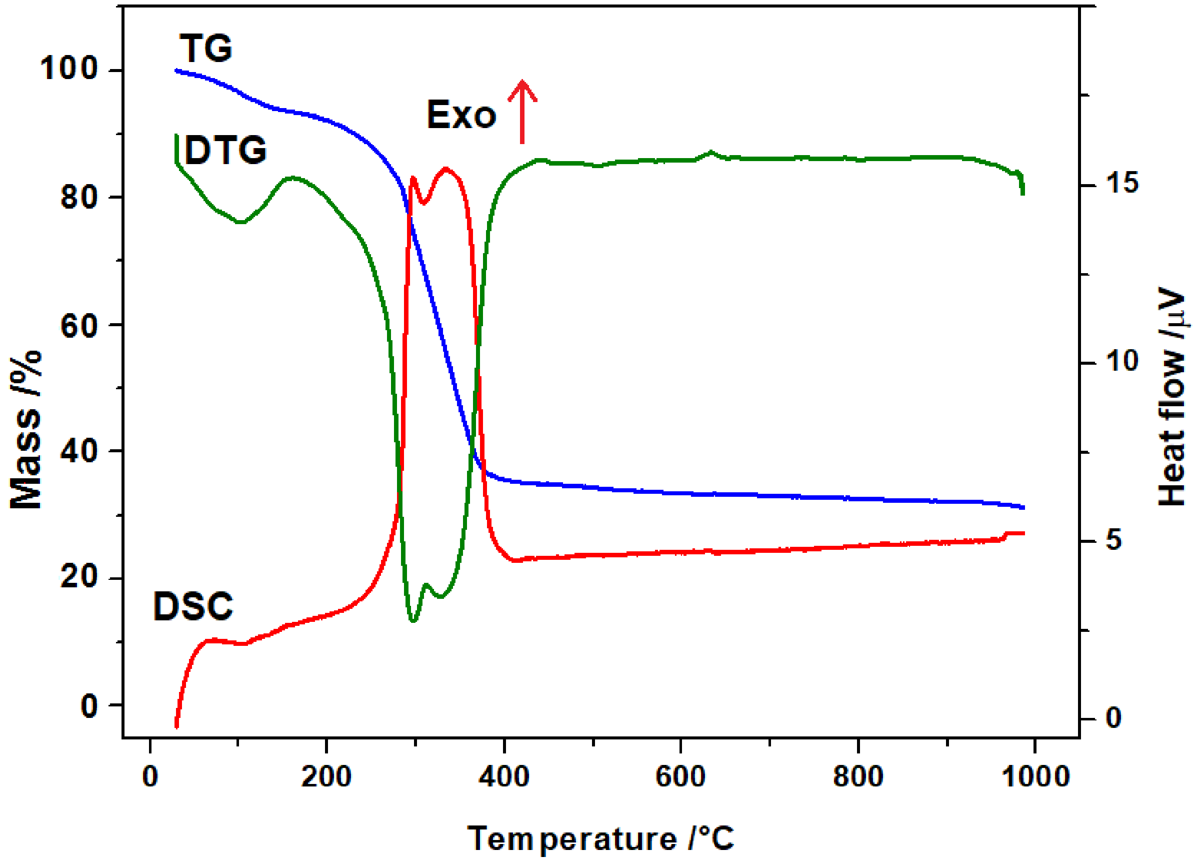

Figure S1. TG, DTG, and DSC curves of Pd^{II}–Eu^{III}–Pd^{II} (**1**) in air.

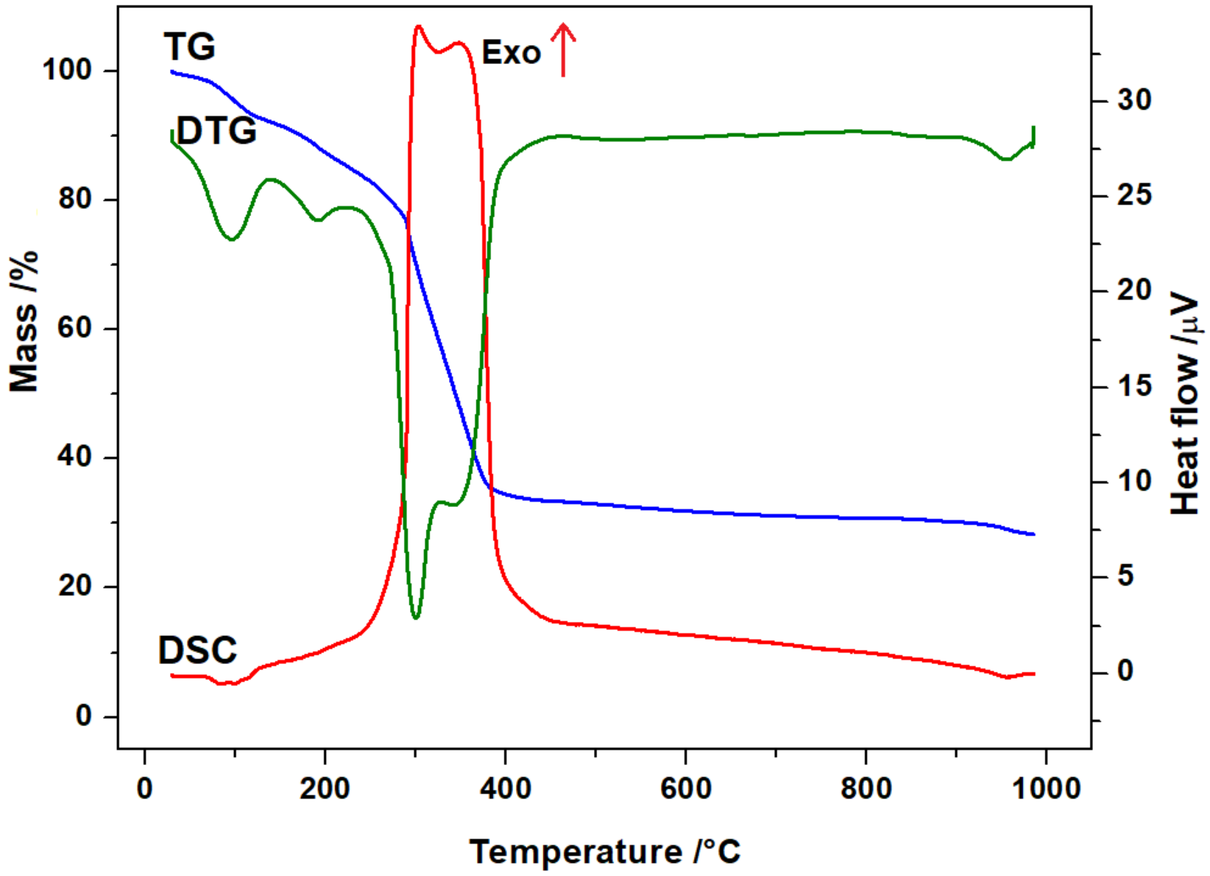

Figure S2. TG, DTG and DSC curves of Pd^{II}–Tb^{III}–Pd^{II} (**2**) in air.

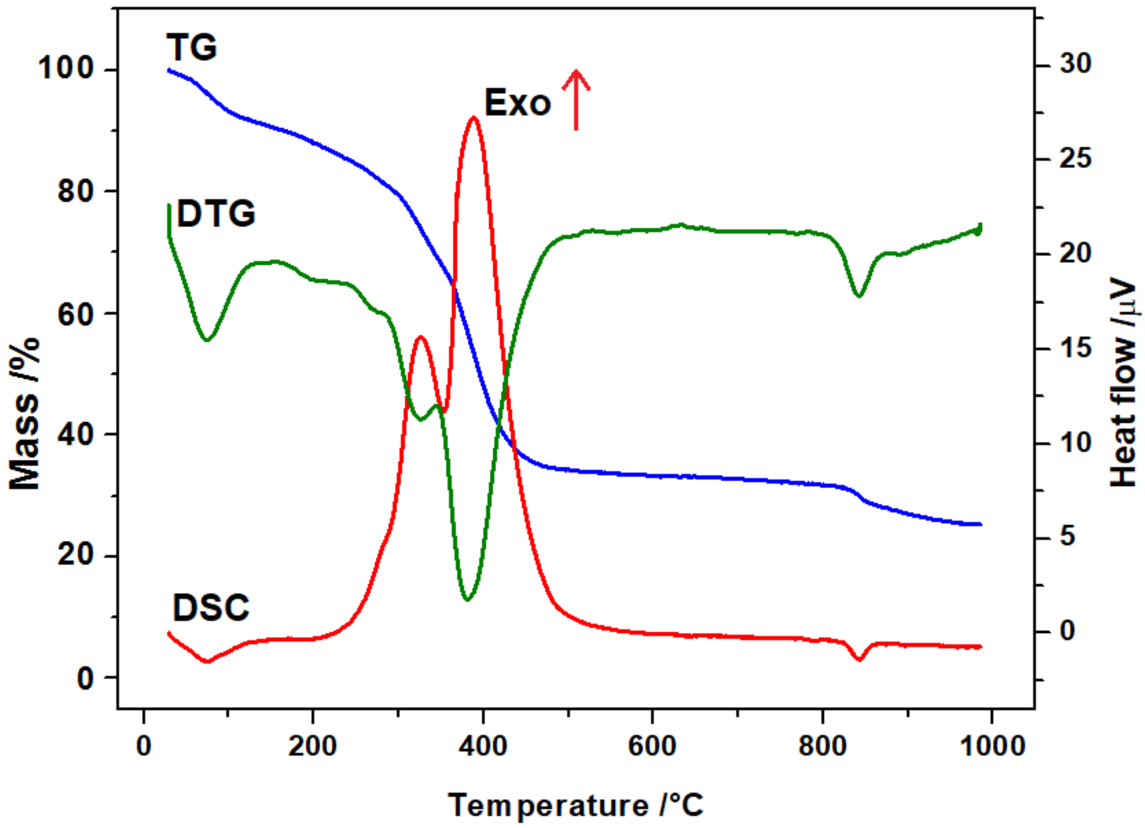

Figure S3. TG, DTG and DSC curves of Pd^{II}–Yb^{III}–Pd^{II} (**4**) in air.

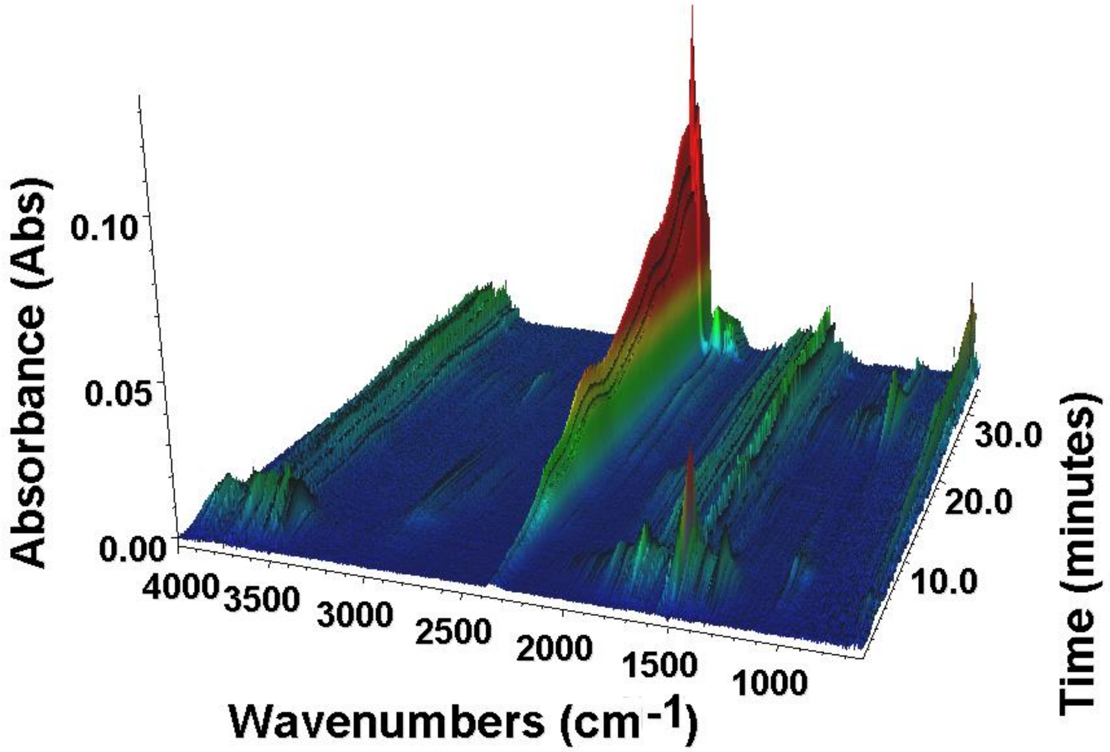

Figure S4. FTIR spectra of gaseous products of the Pd^{II}–Er^{III}–Pd^{II} (**2**) decomposition.

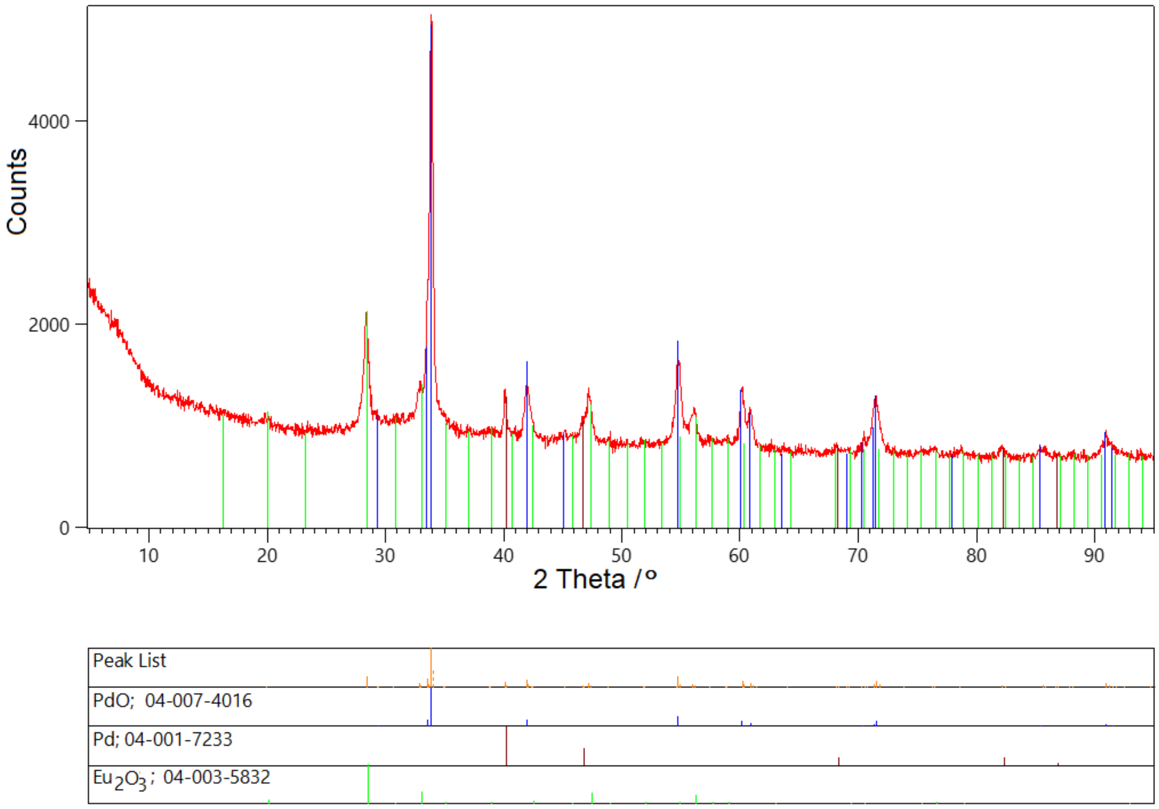

Figure S5. The X-ray powder diffraction patterns of the final products of Pd^{II}–Eu^{III}–Pd^{II} (**1**) decomposition in air.

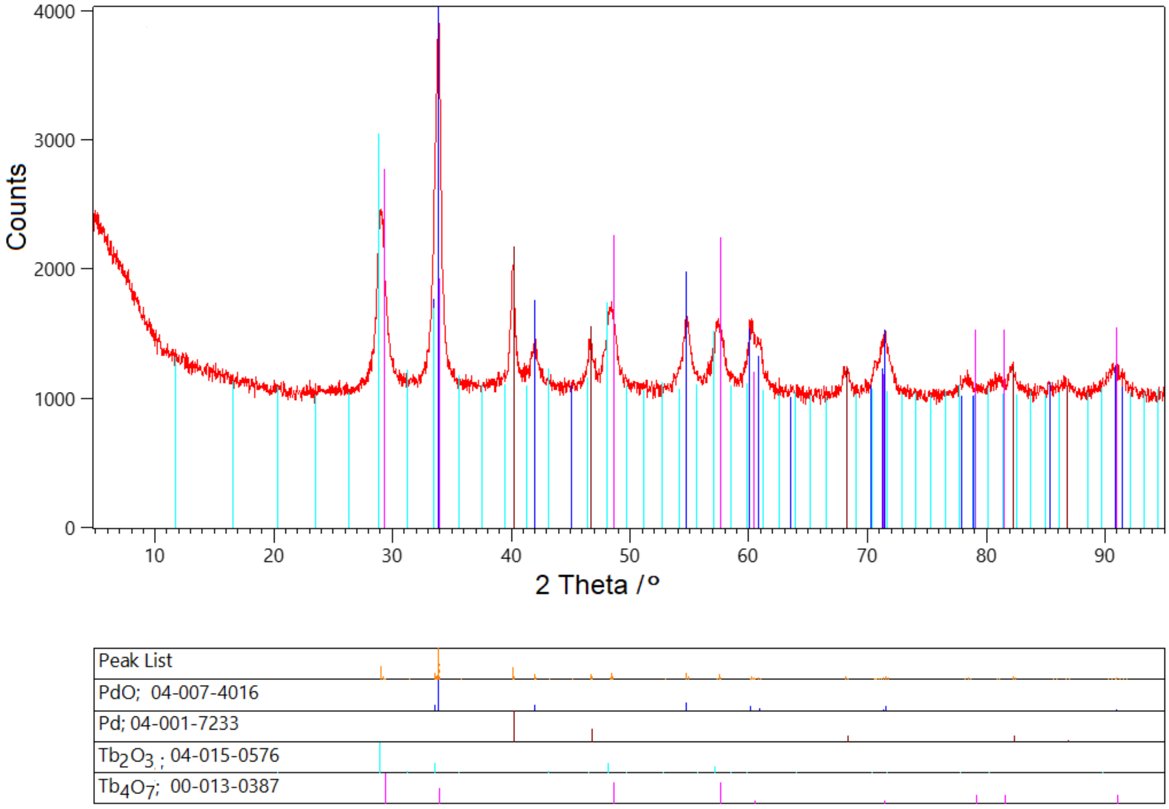

Figure S6. The X-ray powder diffraction patterns of the final products of Pd^{II}–Tb^{III}–Pd^{II} (**2**) decomposition in air.

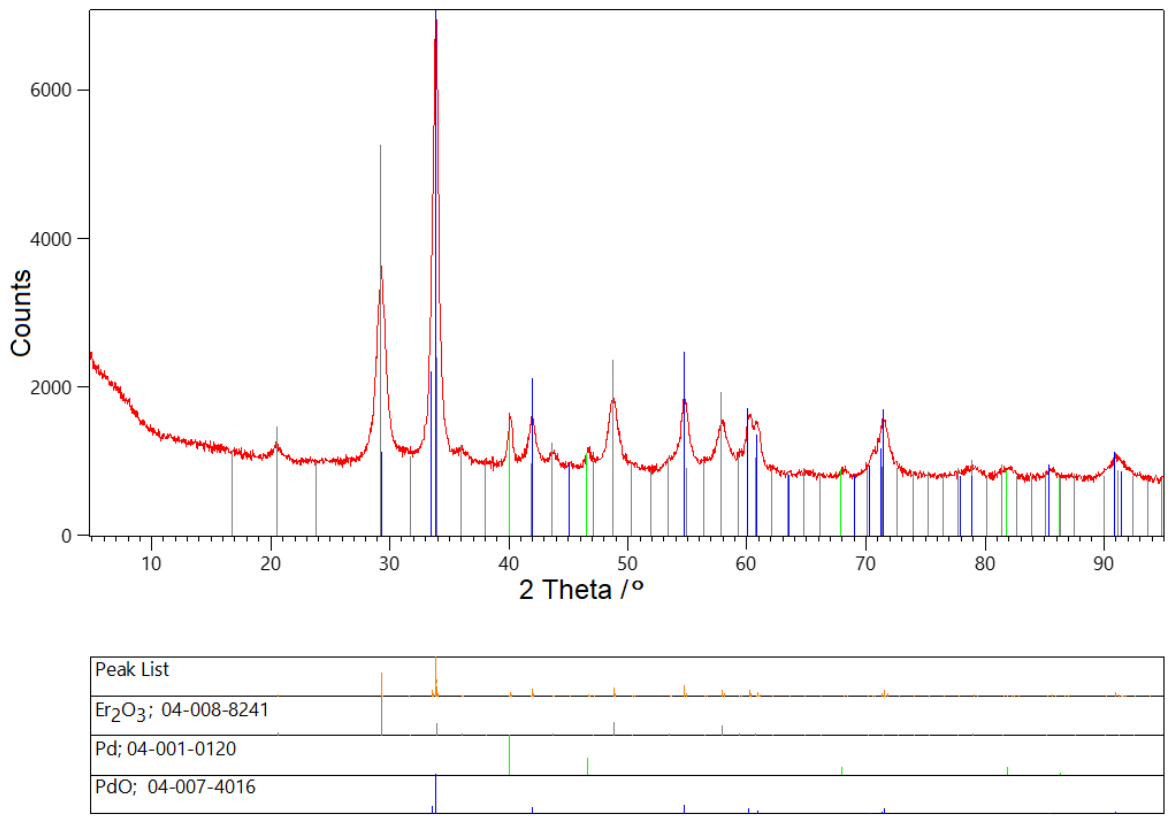

Figure S7. The X-ray powder diffraction patterns of the final products of Pd^{II}–Er^{III}–Pd^{II} (**3**) decomposition in air.

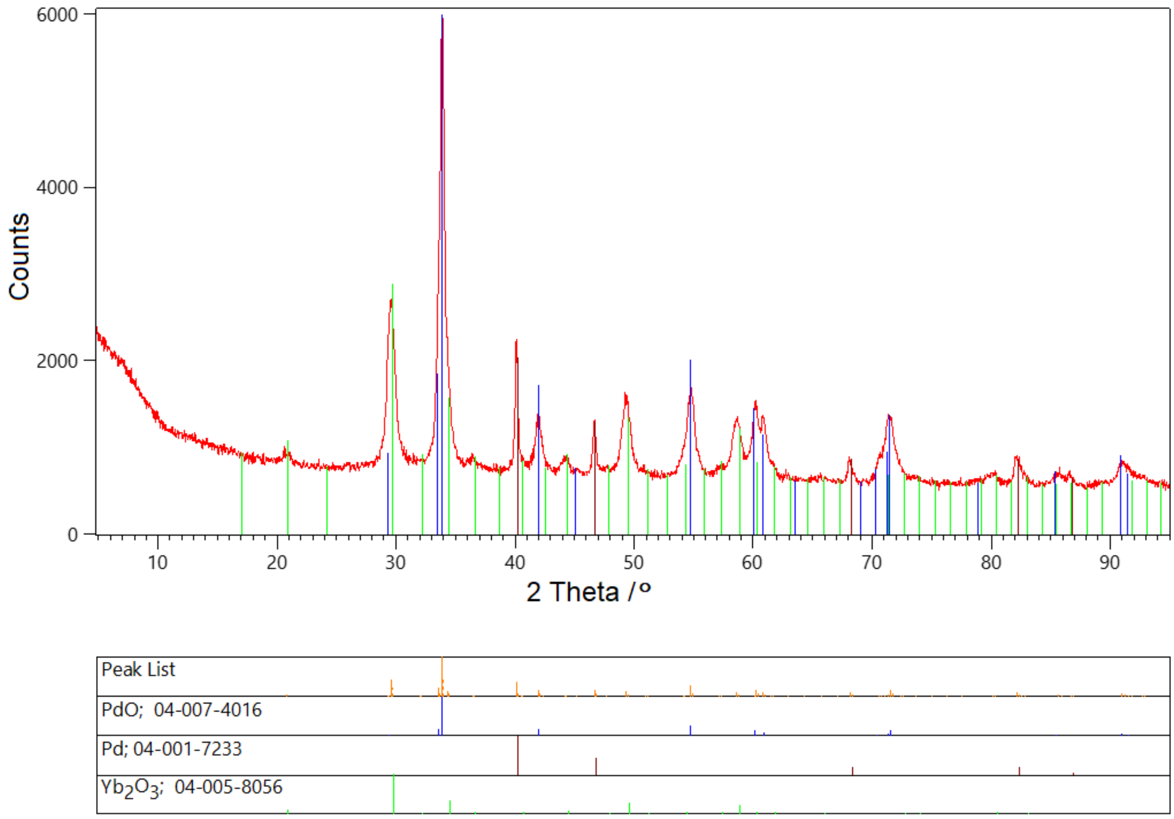

Figure S8. The X-ray powder diffraction patterns of the final products of Pd^{II}–Yb^{III}–Pd^{II} (**4**) decomposition in air.

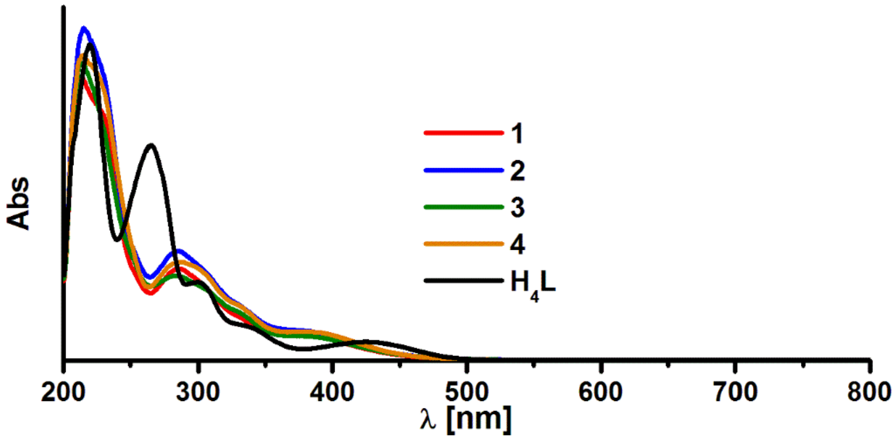

Figure S9. Absorption spectra of Schiff base ligand **H$_4$L** and its metal-mixed complexes **1**-**4** dissolved in methanol (c~2·10^{-5}M).

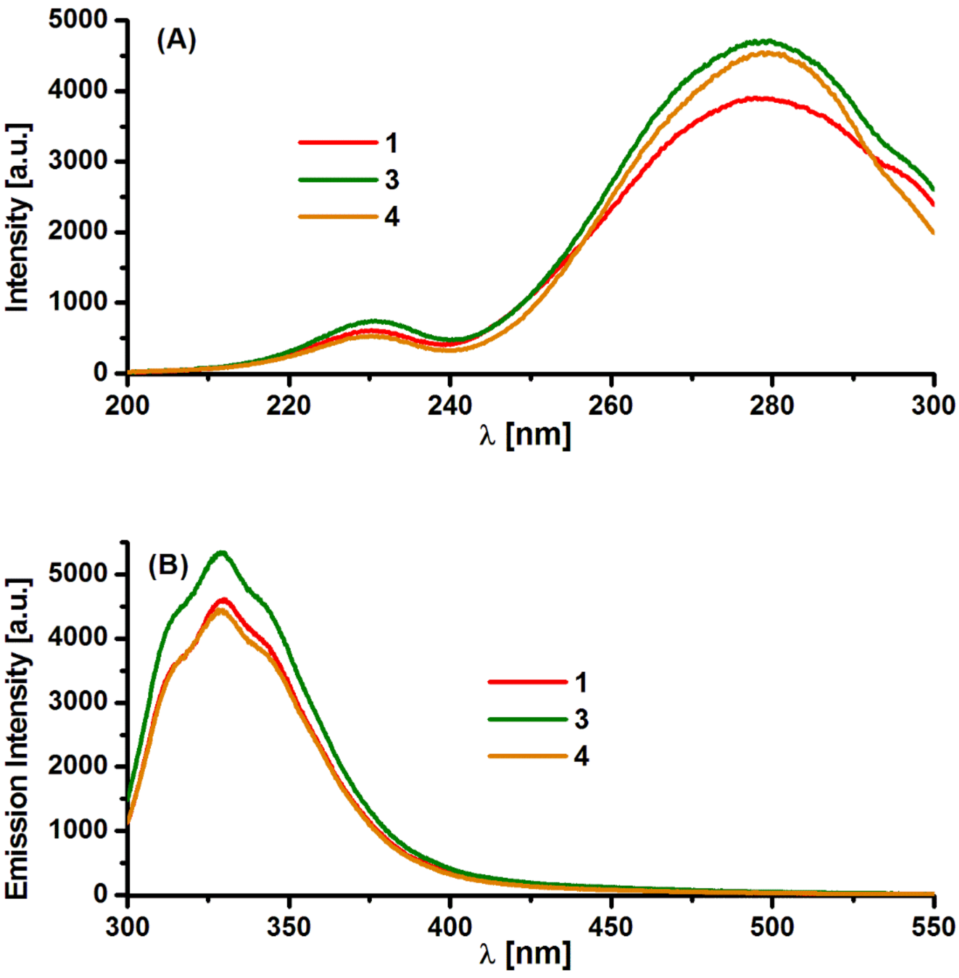

Figure S10. Excitation (A) and emission (B) spectra of methanolic solutions of reported complexes.

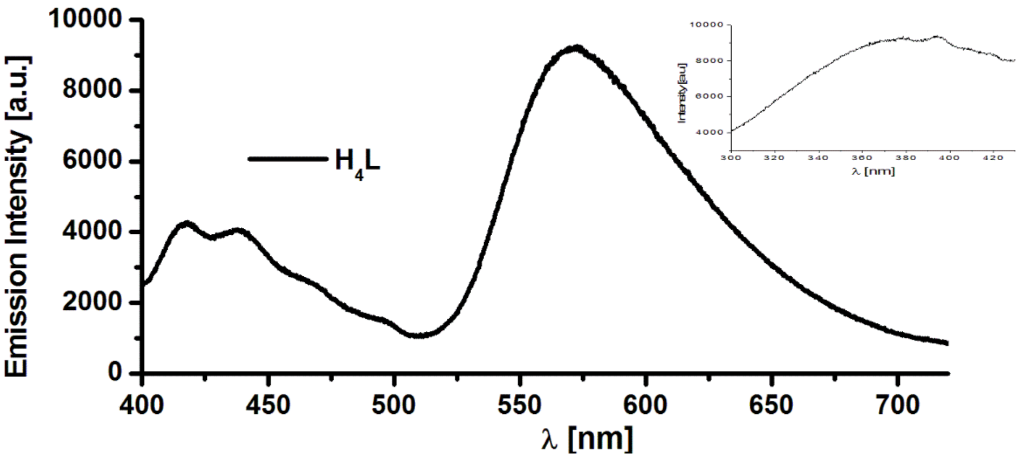

Figure S11. Luminescence spectrum of ligand **H_4L** in solid state; the inset is the excitation spectrum.

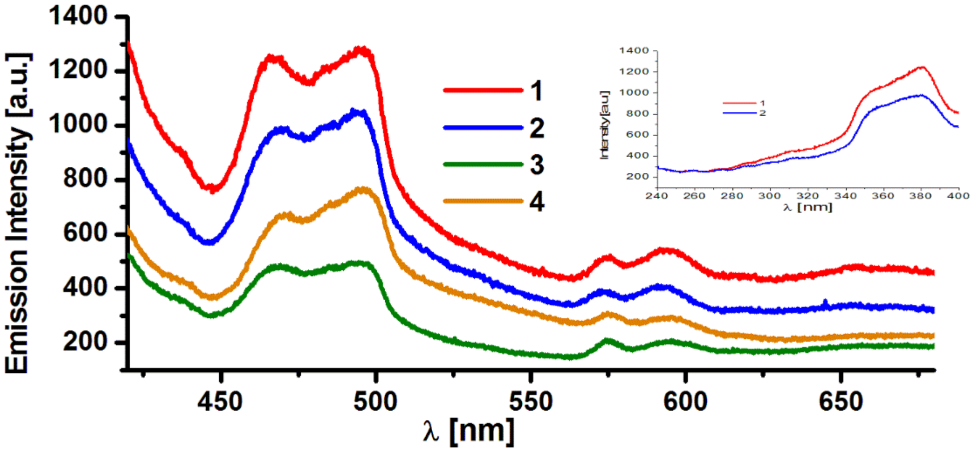

Figure S12. Luminescence spectra of reported complexes in solid state, the inset present the excitation spectra of selected complexes.

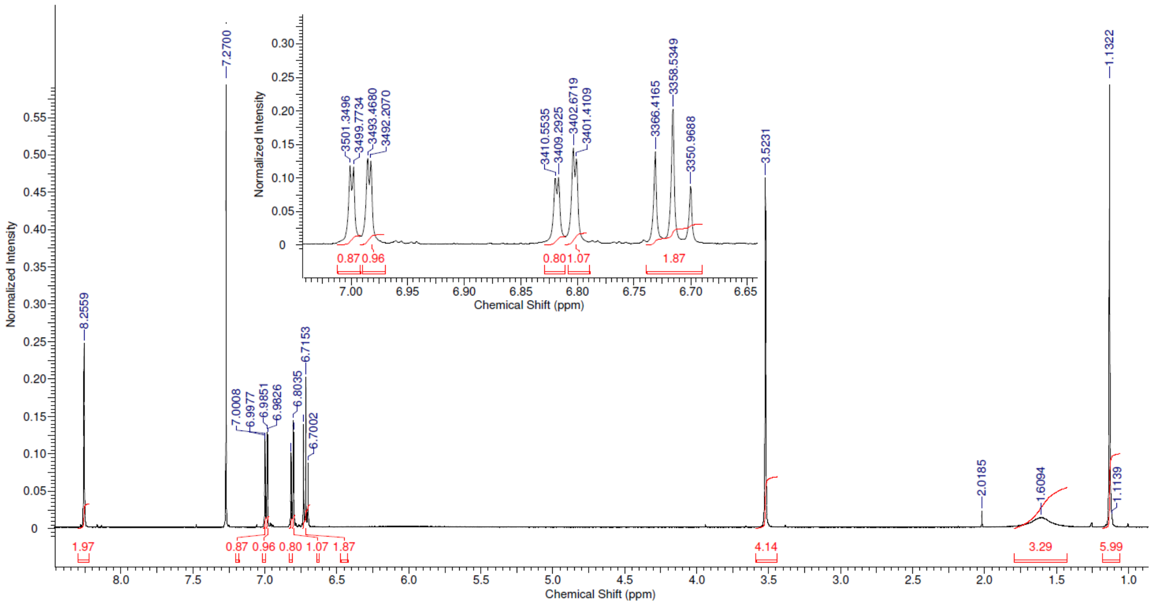

Figure S13. ^{1}H NMR spectrum of ligand **H_4L** in $CDCl_3$ solution.

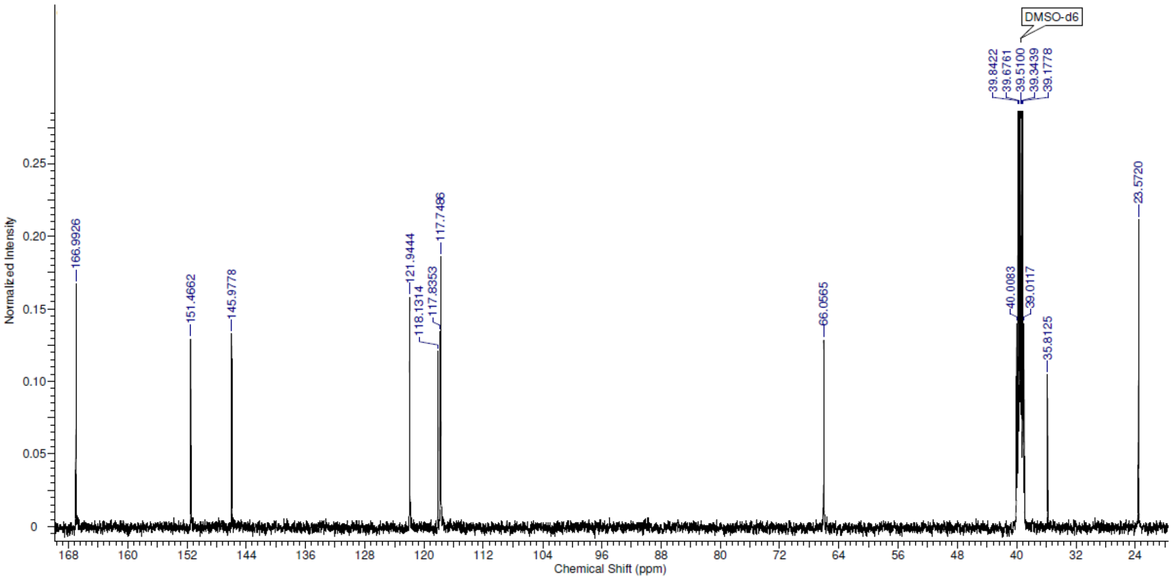

Figure S14. ^{13}C NMR spectrum of ligand **H_4L** in DMSO-d6 solution.
